# The SPECIES beamline at the MAX IV Laboratory: a facility for soft X-ray RIXS and APXPS

**DOI:** 10.1107/S1600577516019056

**Published:** 2017-01-01

**Authors:** Samuli Urpelainen, Conny Såthe, Walan Grizolli, Marcus Agåker, Ashley R. Head, Margit Andersson, Shih-Wen Huang, Brian N. Jensen, Erik Wallén, Hamed Tarawneh, Rami Sankari, Ralf Nyholm, Mirjam Lindberg, Peter Sjöblom, Niclas Johansson, Benjamin N. Reinecke, M. Alif Arman, Lindsay R. Merte, Jan Knudsen, Joachim Schnadt, Jesper N. Andersen, Franz Hennies

**Affiliations:** a MAX IV Laboratory, Lund University, PO Box 118, SE-221 00 Lund, Sweden; b Department of Physics and Astronomy, Uppsala University, PO Box 516, SE-751 20 Uppsala, Sweden; c Division of Synchrotron Radiation Research, Department of Physics, Lund University, PO Box 118, 221 00 Lund, Sweden; d Lawrence Berkeley National Laboratory, 1 Cyclotron Road, Berkeley, CA 94720, USA

**Keywords:** RIXS, APXPS, beamlines, MAX IV

## Abstract

SPECIES is an undulator-based soft X-ray beamline that replaced the old I511 beamline at the MAX II storage ring. SPECIES is aimed at high-resolution ambient-pressure X-ray photoelectron spectroscopy (APXPS), near-edge X-ray absorption fine-structure (NEXAFS), X-ray emission spectroscopy (XES) and resonant inelastic X-ray scattering (RIXS) experiments. The beamline has two branches that use a common elliptically polarizing undulator and monochromator. The beam is switched between the two branches by changing the focusing optics after the monochromator. Both branches have separate exit slits, refocusing optics and dedicated permanent endstations. This allows very fast switching between two types of experiments and offers a unique combination of the surface-sensitive XPS and bulk-sensitive RIXS techniques both in UHV and at elevated ambient-pressure conditions on a single beamline. Another unique property of the beamline is that it reaches energies down to approximately 27 eV, which is not obtainable on other current APXPS beamlines. This allows, for instance, valence band studies under ambient-pressure conditions. In this article the main properties and performance of the beamline are presented, together with selected showcase experiments performed on the new setup.

## Abstract 2

SPECIES, the soft X-ray beamline for resonant inelastic scattering and ambient-pressure photoelectron spectroscopy at MAX IV, is described.

## 1. Introduction   

Beamline I511 on the MAX II storage ring of the MAX IV Laboratory (formerly MAX-lab), which was decommissioned in 2013, combined successfully two types of spectroscopies: X-ray photoelectron spectroscopy (XPS) under ultrahigh-vacuum (UHV) conditions and resonant inelastic X-ray scattering (RIXS) (Denecke *et al.*, 1999 ▸). The beamline was based on the proven SX-700 monochromator design, and had a spherical focusing mirror that allowed a balance between high photon energy resolution and high flux (Reininger & Saile, 1990 ▸). Owing to the inclined exit beam of the monochromator the experimental stations were placed at a height of more than 2 m above the experimental hall floor. Switching between the two spectroscopy branches was achieved by an additional switching mirror, causing additional reflection losses in the beam intensity. During the last years of operation, changes in the beamline instrumentation set new demands for the beamline: the existing refocusing optics, and especially the fairly short exit arm for the photoemission branch, were not compatible with the new instrument designed for near ambient-pressure photoelectron spectroscopy (Schnadt *et al.*, 2012 ▸). In addition, placing the instrument on an elevated platform resulted in a less stable system compared with a floor-mounted one. On the RIXS branch the development of new spectrometers required a small vertical spot, three to four times smaller than that provided by I511, to take advantage of the new optical designs. The SPECIES beamline was designed to meet the new requirements set by the instrumentation, while still relying on the same experimental techniques, RIXS and XPS; for the latter, the main interest was shifted towards ambient-pressure X-ray photoelectron spectroscopy, APXPS, at pressures up to a few tens of millibars.

The optical design of the beamline is based on a collimated plane-grating monochromator (cPGM), following examples given by successful high-resolution spectroscopy beamlines at BESSY II (Follath *et al.*, 1998 ▸; Jiang *et al.*, 2004 ▸) and SLS (Strocov *et al.*, 2010 ▸). This concept allows a horizontal beam after the monochromator at a reasonable height above the floor (about electron beam height in the ring). The additional switching mirror is rendered obsolete as the switching can be performed by side-deflecting focusing mirrors. The refocusing solutions for both branches are designed in such a way that the beam is horizontal at the experiments.

Owing to the increased demand for circularly polarized light, the new beamline has an elliptically polarizing undulator as a source, instead of the old planar undulator. The beamline covers an energy range of approximately 27–1500 eV with variable polarization. Even when installed at the aging MAX II storage ring, the conditions for spectroscopy studies were good, especially at low photon energies. Further improvements are expected after the move to the MAX IV Laboratory.

In this paper we outline the main properties and performance of the beamline as characterized during operation at the MAX II storage ring. We will also discuss the experimental possibilities and present a few showcases of experiments which have been performed on the new beamline. Future plans and upgrades after installation at the 1.5 GeV electron storage ring at the MAX IV Laboratory are highlighted.

## 2. Beamline overview   

### 2.1. Source and common optics   

The source for the SPECIES beamline is an elliptically polarizing undulator of APPLE-II type (Sasaki *et al.*, 1993 ▸) manufactured in-house. The insertion device is called EPU61 and it has 41.5 periods and a period length of 61 mm. It has a frame made of cast iron and the magnets are glued together in pairs in order to minimize the mechanical deformation of the frame and magnet holders during phase shifts. The maximum radiated power is ∼720 W at MAX II storage ring. The estimated maximum flux into the beamline is ∼10^15^ photons s^−1^ into a 0.1% bandwidth. The insertion device was tuned and characterized in a magnetic bench prior to installation at the MAX II storage ring, where the vacuum chamber is 15 mm thick and the minimum gap restricted to 16.5 mm. The RMS phase error for the planar and vertical modes of operation are less than 2.2° over the full gap range 14–200 mm. The EPU61 undulator will be characterized at the new magnetic measurement laboratory prior to installation at the MAX IV Laboratory 1.5 GeV storage ring. In addition, two corrector magnets will be fabricated and installed flanking the undulator to compensate for orbit disturbances due to the changing gap and phase in a feed-forward scheme. The new vacuum chamber has an outer vertical aperture of 12 mm allowing operation over the full gap range.

EPU61 is equipped with passive L-shaped shims of soft magnetic material in order to compensate for the dynamic multipoles that appear in the helical and vertical mode of operation (Chavanne *et al.*, 2000 ▸). Commissioning at the MAX II storage ring showed that the device can be operated without disturbing the lifetime of the electron beam too much, except for when very small gaps are used for producing the 45° inclined mode. This is the expected performance of the passive L-shim method, which is unable to compensate fully for the dynamic multipoles in the incline mode of operation. This problem will be addressed at the MAX IV Laboratory by using extra coils in the sextupole magnets flanking the undulator to drive the skew quadrupole field for compensation in a feed-forward scheme.

**Figure 1:**
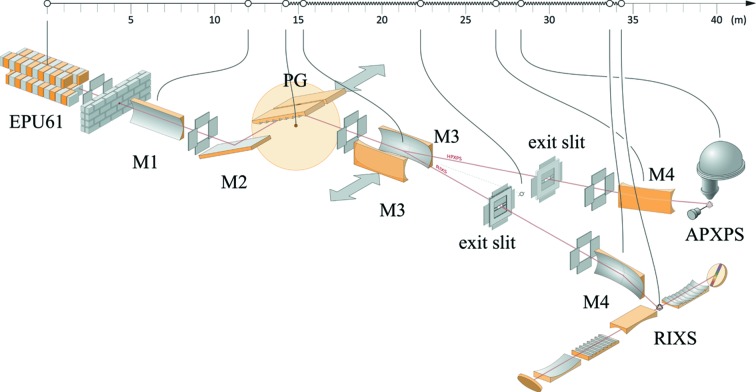
Beamline layout of the SPECIES beamline (courtesy of Johnny Kvistholm). The first mirror (M1) is cylindrical, collimating the beam vertically, whereas both the focusing mirrors (M3) and the refocusing mirror (M4) for the APXPS branch are toroidal. The refocusing mirror (M4) of the RIXS branch is ellipsoidal (rotational ellipse).

The beamline utilizes a plane-grating monochromator illuminated with collimated light (cPGM) (Follath *et al.*, 1998 ▸) for energy selection. The beamline is split into two branches, one of them dedicated to RIXS and the other to APXPS, by using two different focusing mirrors after the grating and both of the branches have their own exit slits. The main constraints of the geometry of the beamline are imposed by the available space at the old MAX II storage ring and the requirements by the RIXS branch, in which both a good photon energy resolution and a small spot are required. The layout of the beamline is presented in Fig. 1 ▸.

The monochromator houses two blazed gratings: an Au-coated one with a ruling density of 1221 lines mm^−1^ and a Ni-coated one with a ruling density of 250 lines mm^−1^. The Ni-coated grating provides a gain in flux with modest resolution in, roughly, the 200–600 eV photon energy region. Both gratings were transferred from the SX-700 monochromator used at the old I511 beamline as there were no manufacturers for blazed gratings during the procurement phase of this beamline. The Au grating was cleaned from carbon contaminations using UV light-generated ozone under ambient conditions prior to installation. The pre-mirror in the monochromator is internally cooled. In fact, side-cooling would have been sufficient at the MAX II storage ring, but since the beamline was designed to be transferred to the 1.5 GeV ring at the MAX IV Laboratory, and will be subjected to a much higher heat load there, an internal cooling scheme was chosen. All the beamline components were built by FMB Berlin except for the gas absorption cell which was made in-house and based on that designed at Paul Scherrer Institute (Schmitt, 2013 ▸).

For high-resolution cPGM beamlines a stigmatic focusing onto the exit slit is beneficial [for details see, for example, the discussion by Strocov *et al.* (2010 ▸)]. Actually, using the first mirror for horizontal focusing results in the highest limit for resolution but at the same time the demagnification to the exit slit is reduced, resulting in a larger spot at the RIXS instrument. Therefore, the focusing mirror also provides the horizontal focusing in the case of the RIXS branch. A stigmatic focus at the RIXS exit slit allows a single, ellipsoidal re­focusing mirror to be used for that branch. The photon energy resolution requirement for the APXPS branch is not so stringent and, in addition, the refocusing mirror is toroidal and thus does not require a stigmatic image at the exit slit either. The refocusing stages and their properties are described in more detail below.

### 2.2. Refocusing stages   

The needs set by the APXPS and RIXS experiments are very different: for RIXS a small, high photon density spot is desired whereas for the APXPS the area accepted by the spectrometer aperture should preferably be nearly completely illuminated to reduce the sample damage by radiation. The spectrometers at both branches are preferably mounted horizontally. This facilitates easier alignment of the instruments and simpler sample manipulator design. These requirements exclude conventional Kirkpatrick–Baez pairs based on plane elliptical mirrors, which can be obtained in high quality. The simplest solution for providing a horizontal, focused beam is to use side-deflecting toroidal, or ellipsoidal, mirrors. This solution also reduces the reflection losses compared with a solution based on a Kirkpatrick–Baez pair.

An ellipsoidal mirror was chosen for the RIXS branch, although at very low photon energies the image at the focal plane shows a hint of bow-tie shape: the ray-tracing simulations showed that this does not compromise the energy resolution of the spectrometer. With slope errors of 0.9 and 2.9 arcsec (tangential and sagittal, respectively) the image remains below the required value even when large exit slit openings are used. The benefit of the side-deflecting refocusing mirror is evident: for vertical imaging there is a forgiveness factor of sin(2°) (Cash, 1987 ▸), and the sagittal slope error has a negligible contribution to the vertical image size. Most of the refocusing mirrors for the MAX IV Laboratory soft X-ray beamlines are also ellipsoidal, based on the same argument.

The requirement of a constant spot size at the APXPS branch, independent of photon energy or exit slit opening, demands a more unconventional optical configuration. It can be met by defocusing and controlling the beam waist at the sample location by monochromator magnification (Grizolli *et al.*, 2013 ▸). One way of realising this would be by decoupling horizontal and vertical focusing using a Kirkpatrick–Baez pair using an adaptive mirror for vertical focusing. This would compensate for the changes in the object size due to different exit slit openings. However, it is also possible to align the toroidal mirror in such a way that the horizontal focus is at the sample while the vertical focus is some tens of millimeters further downstream (astigmatic focusing). By doing so the vertical beam size becomes more dependent on the divergence of the source rather than its size. In the case of a cPGM monochromator, the vertical divergence at the exit slit plane can easily be controlled by the monochromator settings. This solution allows a single, non-adaptive refocusing mirror, yet providing close-to-constant (vertical) image size at the sample plane independent of the exit slit opening or photon energy. The principle of astigmatic focusing was also tested during the commissioning, and the vertical beam size at the sample was found to be constant, and very close to the expected 100 µm (FWHM).

### 2.3. Beamline control   

SPECIES, being the first beamline to use the standard MAX IV control system, served also as a prototype platform for developing and setting the soft X-ray beamline motion control standard at the MAX IV Laboratory. Here we give a brief description of the control system. A more detailed discussion can be found by Sjöblom *et al.* (2016 ▸).

The SPECIES beamline has 56 motorized axes on the beamline and 22 axes on the endstations. The 56 beamline axes were integrated into the control system at the MAX II storage ring, and the remaining 22 axes will be integrated at the new MAX IV Laboratory. The standard motion controller used at the MAX IV Laboratory is the IcePAP motion controller (Janvier *et al.*, 2013 ▸) developed at the ESRF. It controls all of the axes in one system. All motorized axes, except that of the monochromator, are run by stepper motors and the motion is monitored by a set of linear absolute and incremental encoders.

The control system is built in Tango (TANGO, 2015 ▸) and uses a Python-based Sardana framework (Coutinho *et al.*, 2011 ▸) to communicate with the IcePAP controllers. At the MAX II storage ring, scans and data acquisitions, as well as other functionalities, were run through a set of Taurus GUIs using Sardana, while a graphical synoptic GUI on top of Sardana and Taurus was developed to be used at the MAX IV Laboratory.

**Figure 2:**
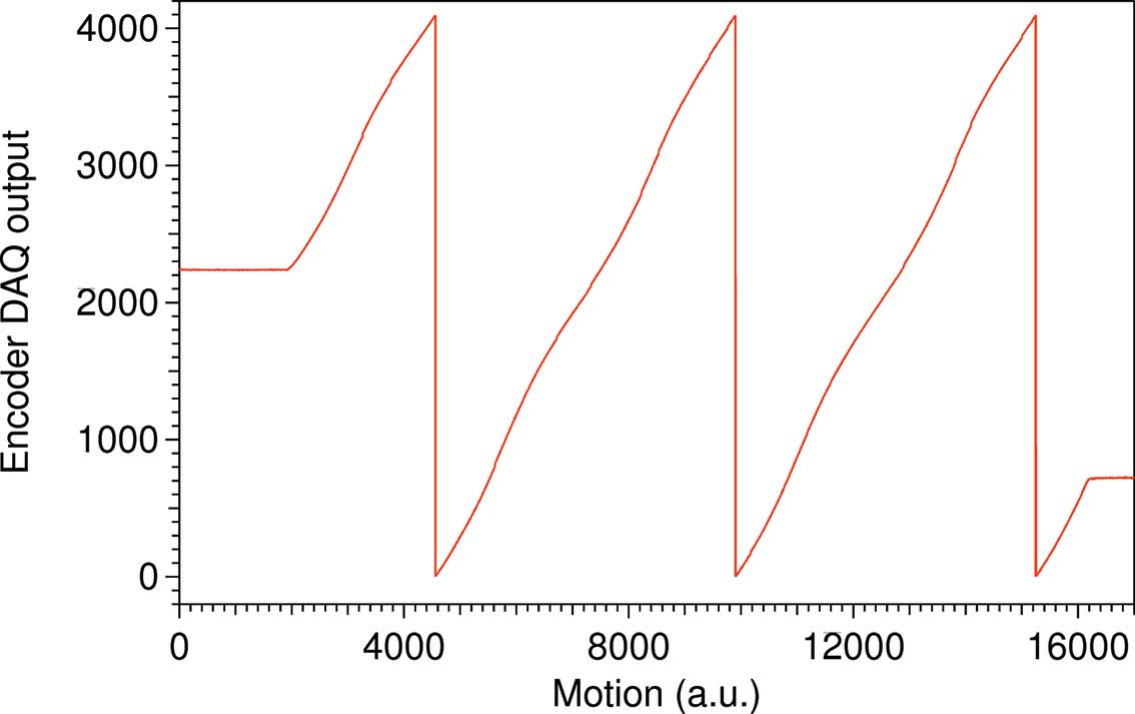
The sinusoidal cyclic error in the sawtooth form of the encoder output that indicates a need of the Heydemann compensation. As the motion progresses, the encoder DAQ should give a linear output from 0 to 4096 and then roll over to create a sawtooth pattern. Instead of a perfectly linear form, there is a periodical error that repeats itself for every sawtooth visible in the plot for one encoder head. Each individual encoder head has its own unique pattern. As a consequence, each DAQ value needs a small correction. As the encoder DAQ is a measure of the mirror and grating pitch angles, and hence the photon energy, it is the energy scale that needs stretching and compressing in the form of Heydemann compensation to correct for the errors.

The SPECIES monochromator uses Heidenhain RON905UHV angular incremental encoders for the mirror and the grating. Each encoder has four individual analog encoder heads mounted at 90° angle with respect to each other. To process the signals, two IK220 counter cards are installed into two PCs, as one card takes signals from two encoder heads. The analog encoder heads create small built-in cyclic errors. As the heads move across one encoder line two sine-shaped 10 µA_pp_ analog currents phase shifted by 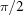 are produced. The errors are visible when the currents are plotted against each other. Instead of a perfect circle, the graph is a tilting ellipse with an offset from the origin. As the analog input to the IK220 cards has a cyclic error, the ADC encoder output also has a cyclic error as shown in Fig. 2 ▸, where the encoder output is not a perfect sawtooth form. Ideally the DAQ output should increase from 0 to 4096 in a linear fashion and then roll over as the motion progresses. Instead of linear, each sawtooth possesses the same wave-like deviation from a straight line. As a result the same DAQ value from each sawtooth needs the same compensation regardless of the photon energy. As each encoder has four individual encoder heads, the error could, to some extent, be averaged out in software by the PC. However, it is not currently possible to send the corrected encoder value back to IcePAP. Instead, analog signals from one of the four encoder heads are forwarded from the IK220 card to an EXE 660B interpolation and digitizer unit.

**Figure 3:**
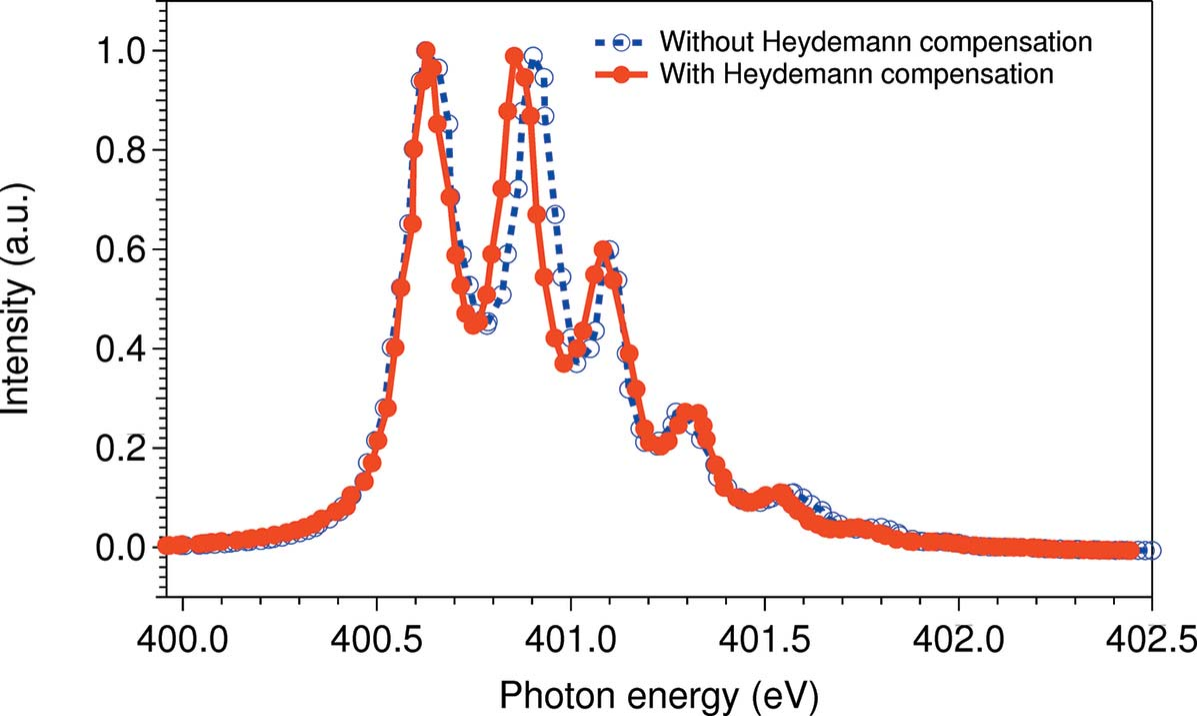
Ion yield spectrum at the N 1*s* absorption edge in N_2_. The blue dashed line shows the measured spectrum without the applied software compensation and the red solid line shows the Heydemann compensated spectrum. The spacing between observed adjacent vibrational levels varies between approximately 236 and 208 meV after compensation.

The EXE 660B produces 400 TTL SSI encoder pulses in every period of analog input signal and these are then forwarded to the IcePAP units. In this setup the encoder errors are fed into IcePAP. The solution to the cyclic error is a Heydemann correction (Heydemann, 1981 ▸; Follath & Balzer, 2010 ▸) in the Sardana layer. With help of the Heydemann compensation the cyclic error is mapped and the angle output is corrected. Also, when a motion request is sent, the target position is corrected. In the end, the cyclic encoder errors correspond to an energy deviation, and in the 398–402 eV span the maximum error was determined to be approximately 47 meV. The result of introducing the Heydemann correction is shown in Fig. 3 ▸, where an N_2_ absorption scan is shown with and without applied correction. The spacing between the adjacent peaks ranges from approximately 236 to 208 meV after the compensation. This is in good agreement with previous studies [see, for example, Chen & Sette (1989 ▸)].

For the majority of the axes, including those of the monochromator, the IcePAP drivers operate in hardware position closed loops, which make the motors compensate any deviation from a defined position read by the corresponding encoder. In the case of the monochromator, additional care is taken to overcome the non-linear relationship between the angular encoder and the stepper motor operating a sine bar, as the movements otherwise may be slow and inaccurate. During commissioning, measurements showed a resolution of 0.5 µrad for the mirror and 0.3 µrad for the grating pitch motions. The smallest resolvable one-step movement in closed loop, including the settling time, takes 0.1 s for the mirror and the grating.

## 3. Ancillary facilities   

The ancillary facilities at the old MAX II storage ring consisted of a room dedicated to sample mounting and UHV equipment preparation and a basic chemistry laboratory for sample preparation. The new MAX IV Laboratory also provides such facilities and chemistry laboratory spaces as well as some storage space for smaller user equipment.

## 4. Facility access   

The SPECIES beamline is expected to be operational again during late 2017. The beamline will be accessible to non-proprietary users by submitting an application in connection to a call for beam time applications. The applications will be peer-reviewed and beam time will be allocated based on the scientific merits and feasibility of the proposed experiments by the program advisory committee. The beamline is also in a limited fashion accessible to proprietary research.

## 5. Highlights   

### 5.1. Beamline performance   

The beamline was built using a laser tracker coordinate system, which relies on fixed target nests around the beamline location. The initial alignment using this coordinate system gave a good starting point for alignment with light, and with small tuning the first mirror of the beamline was brought onto the axis of undulator radiation.

**Figure 4:**
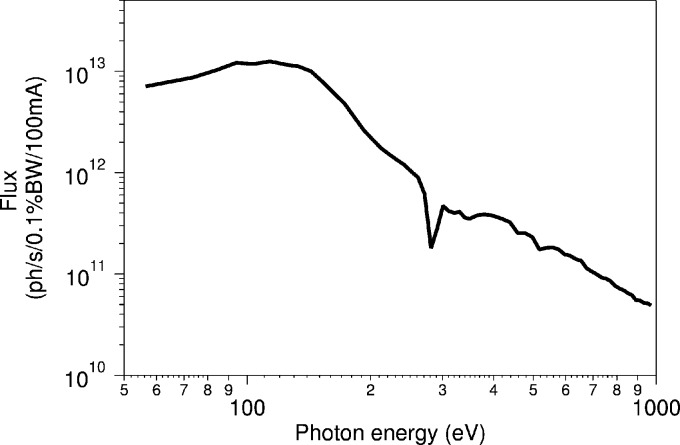
Measured flux after the exit slit on the APXPS branch. The flux curves were measured with a beam-defining aperture (after the collimating mirror) opening of 0.5 mm × 0.5 mm corresponding to approximately 0.04 mrad × 0.04 mrad.

The commissioning measurements and experiments performed at the SPECIES beamline show that the photon flux at experiments is very close to calculated values (*cf.* Table 1 ▸). A flux measurement performed using an IRD AXUV100 photodiode located after the APXPS branch exit slit is presented in Fig. 4 ▸. The flux curve was measured into a very small angular opening of approximately 0.04 mrad × 0.04 mrad controlled by a beam-defining aperture after the collimating mirror. The exit slit opening was controlled throughout the scanning in order to keep the bandwidth at 0.1%.

**Figure 5:**
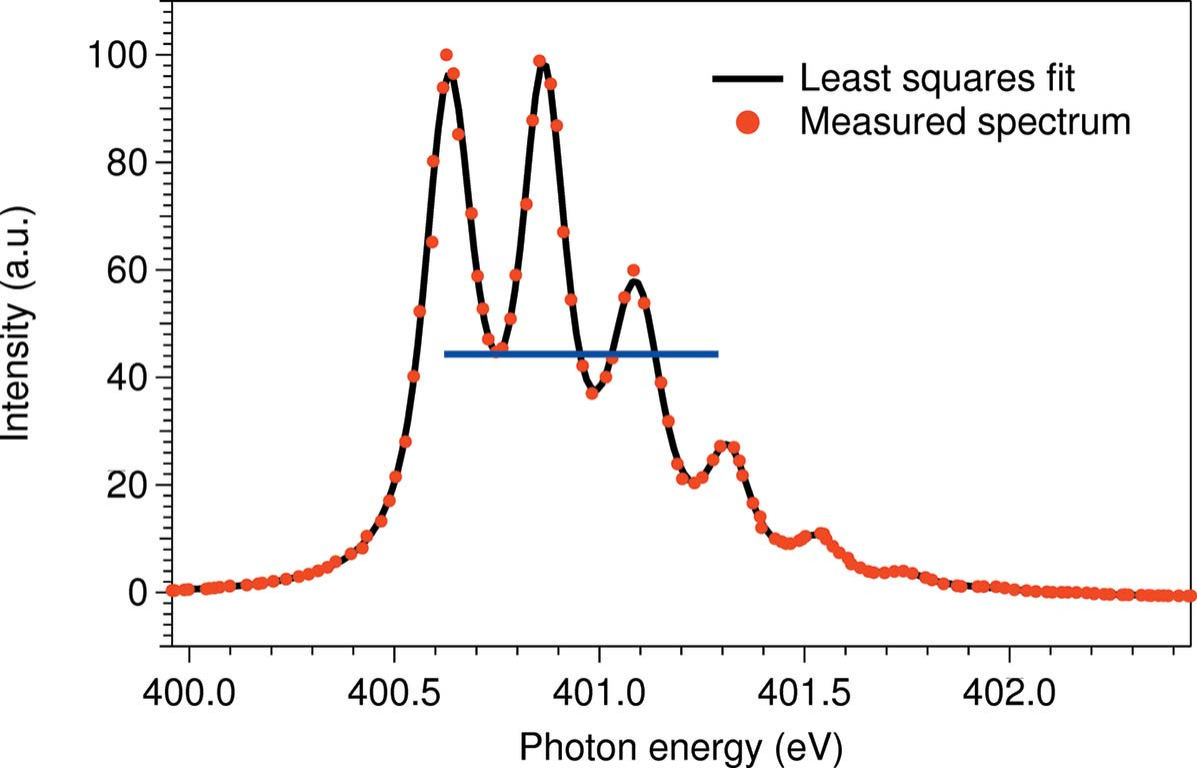
Ion yield spectrum at the N 1*s* absorption edge in N_2_. The blue line is to emphasize the first valley and the third vibrational peak: this ratio reflects the experimental resolution. The black curve shows the result of the least-squares fit with a Lorentzian width of 120 meV and a Gaussian width of 50 meV.

The photon energy resolution of the beamline was measured by studying the absorption spectra of N_2_ molecules recorded with a gas cell. The ion yield spectrum at the N_2_ 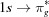 excitation region is shown in Fig. 5 ▸. Due to the relatively large lifetime broadening of the excited state, the evaluation of the photon energy resolution is difficult by direct deconvolution. A least-squares fit gives a Lorentzian width of 120 meV and a Gaussian width of 50 meV, which corresponds to a resolving power of 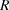 = 8000. However, using the method presented by Chen & Sette (1989 ▸), the instrumental broadening can also be estimated more reliably by calculating the ratio between the intensities of the first valley in the spectrum and the third peak. The ratio determined here gives, according to Chen & Sette (1989 ▸), a Gaussian broadening of ∼30–40 meV, or better, resulting in a resolving power 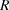 > 10000. We therefore conclude that the monochromator reaches the design criteria of reaching a resolving power of 10000.

The energy scale of the monochromator was calibrated using the method that has been succesfully used at BESSY for calibrating plane-grating monochromators (Weiss *et al.*, 2001 ▸). For this the same N_2_ absorption spectrum as for the resolving power determination was recorded for different fixed focus constants of the monochromator. Using this method the energy of the first peak of the resonance has been determined to be 400.63 eV. At the same time the relative energy shifts have been observed to be smaller than 10^−4^.

**Figure 6:**
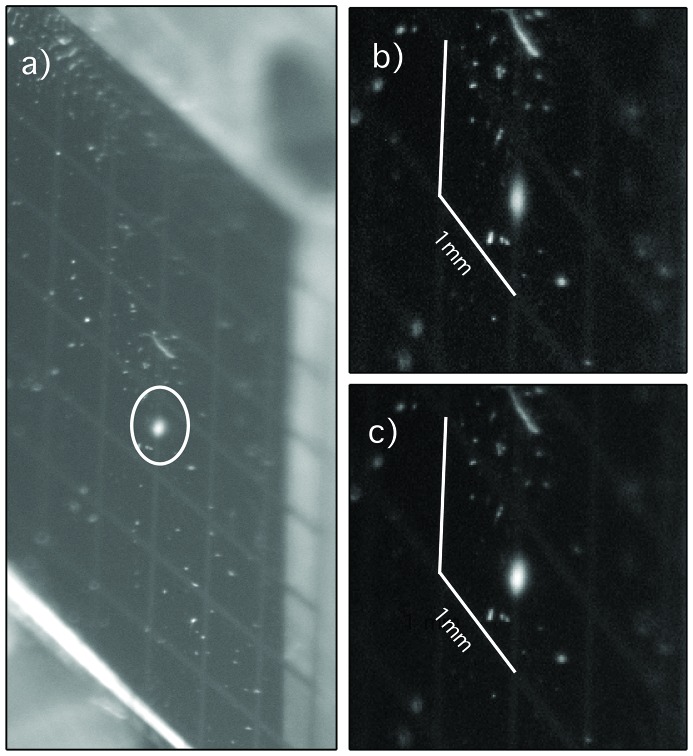
Spot at the APXPS endstation sample position captured at a photon energy of 250 eV. Panel (*a*) shows the Ce:YAG crystal mounted with adhesive carbon tape on a sample holder of the APXPS system. The beam spot is circled. Panels (*b*) and (*c*) show the beam spot at the photon energy of 250 eV and *c*_ff_ = 2.25 for exit slit openings of 500 µm and 50 µm, respectively. The intensity of the photon beam was attenuated by a 200 nm-thick Al window for recording the spot size with the larger slit opening in order to keep the saturation of the YAG crystal at minimum and to be able to compare the spot sizes. The white lines in panels (*b*) and (*c*) show the dimensions of the image (1 mm).

The effect of using astigmatic focusing at the APXPS branch was studied during commissioning by recording the images of the synchrotron radiation beam spot on a Ce:YAG crystal mounted onto a sample plate using adhesive carbon tape. The images are presented in Fig. 6 ▸. The images were captured at the photon energy of 250 eV at two significantly different slit sizes of 50 µm and 500 µm. In order not to saturate the image, the beam intensity was kept approximately constant (monitored by a drain current measurement from the refocusing mirror) by attenuating it with a 200 nm-thick Al window, when using the 500 µm exit slit opening. In the case of stigmatic focus the beam size should be ten times larger at the larger slit conditions, but the images show that the beam size is only marginally affected by the exit slit opening. This indicates that the astigmatic focusing scheme can indeed be used to make the beam size independent of the exit slit opening at the sample position. Furthermore, the images show that the beam sizes match well with the expected 100 µm (FWHM).

At MAX IV 1.5 GeV storage ring the horizontal spot size and maximum photon energy resolution of the beamline will improve compared with values reached at MAX II storage ring. This follows from the horizontal and vertical source sizes being approximately 50% and 30% smaller, respectively, at the new storage ring. The quality of the optical components was specified in such a way that their effect remains negligible also at the new storage ring. For resolving powers beyond the target value, which might otherwise be achievable, the slope errors of the old grating start to limit the performance.

### 5.2. RIXS endstation   

The RIXS endstation has two grating spectrometers for RIXS measurements. Together these cover the energy range from 27 to 1500 eV. The two spectrometers are mounted horizontally, facing each other, perpendicular to the photon beam (see Fig. 1 ▸). The low-energy region of the beamline, 27–200 eV, is covered by a plane-grating spectrometer, PGS (Agåker *et al.*, 2009 ▸), whereas a modified grazing-incidence Rowland circle spherical-grating spectrometer, GRACE (Nordgren *et al.*, 1989 ▸), covers the energy range 50–1500 eV. In addition to the two spectrometers, a retractable detector for NEXAFS measurements is also available at the endstation.

Both of the RIXS instruments take advantage of the small vertical beam size at the focal plane, imaging the beam spot directly onto the detector without any entrance slits. They are both equipped with multichannel plate delay line detectors which makes it possible to synchronize the detectors to the synchrotron bunch structure. This allows a gating scheme to be implemented, increasing the signal-to-noise level as the synchrotron radiation repetition rate is relatively low compared with the detection time which is in the nanosecond range. To further increase the efficiency of the detectors the front plate is enhanced for UV and X-rays by a coating with a high photo-yield material, such as caesium iodide.

The resolving power of the PGS instrument, 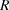 ≃ 7500 at 75 eV, matches very well with the achievable resolving power of the beamline (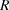 > 10000). The performance of this instrument was tested at BESSY II during a number of commission beam times before being moved to MAX IV Laboratory. The use of a collimating mirror before the grating gives a relatively high acceptance angle, 5000 mrad^2^, resulting in reasonable counting rates even with high resolution and the increased number of reflections. The modified GRACE instrument was initially designed much more for covering a wide photon energy range with reasonable resolution (*R* few thousands) and acceptance (around 1000 mrad^2^), but it has been shown to be a very versatile tool for RIXS experiments at synchrotron radiation sources (Kuusik *et al.*, 2013 ▸; Magnuson *et al.*, 2012 ▸; Nilsson *et al.*, 2010 ▸), including free-electron lasers (Kunnus *et al.*, 2012 ▸).

The spectrometers at the RIXS station are mounted on a custom chamber, designed for measurements on samples ranging from solids to liquids and the gas phase. The chamber is pumped by two turbomolecular pumps mounted beneath the experimental region. The design also includes a very compact valve structure, where both vacuum and filter valves are co-located between the instruments and the experimental volume, enabling a separation of the vacuum in the spectrometers and the experimental chamber to protect the optics from contamination. Each spectrometer has its own turbomolecular pump to keep vacuum while the instrument is separated from the experimental chamber vacuum. There is also a differential pumping stage and the possibility to insert a foil between the refocusing mirror (M4) of the beamline and the experimental chamber for protecting the vacuum while running high-pressure experiments.

A small load-lock chamber, enabling docking and sample transfer from a vacuum suitcase, is mounted on top of the experimental chamber. The samples are moved within the system by using a compact, large bore four-axis manipulator designed to hold custom sample rods. The motorization of the manipulator enables sample scanning to avoid radiation damage. The scanning function can also be used for spectral raster imaging. The manipulator rod can be fully extracted from the experimental chamber, into the load lock, while a valve can close the passage between the two chambers. This allows for rapid change of the samples or extraction and replacement of the manipulator rod without breaking the vacuum in the main chamber.

### 5.3. APXPS endstation   

The APXPS endstation is described in detail elsewhere (Schnadt *et al.*, 2012 ▸; Knudsen *et al.*, 2016 ▸) and is discussed here only briefly. The endstation is dedicated to *in situ* and *operando* studies of the solid–vapor and liquid–vapor interfaces under ambient-pressure conditions using XPS and NEXAFS using partial and total electron yield detection. Although equipped for ambient (in this context up to a few tens of mbars) pressure studies, conventional UHV studies of surfaces are also possible due to the special dockable and retractable ambient pressure (AP) cell. This allows the users to change between high pressure and UHV studies in a matter of minutes without breaking the vacuum, making it possible to prepare and characterize the surface *in situ* before performing the ambient-pressure experiments. In addition, the AP cell itself can be changed relatively easily allowing studies in different sample environments that might require different configurations in terms of, for example, cell materials, cell volumes and sample geometries. Also user modified and specialized cells are possible to mount on the system. This makes the setup extremely versatile and facilitates studies within several disciplines such as catalysis, electrochemistry, corrosion, solar cells, fuel cells as well as liquid, biological and geological samples.

The endstation is equipped with a SPECS PHOIBOS 150 NAP electron energy analyzer. The analyzer features a special head of the electrostatic lens system, on which the AP cell can be docked using a bayonet-like coupling mechanism. The analyzer can be operated in various magnifications, angular dispersion, transmission and acceleration modes optimized for various conditions such as different spot sizes on the sample. To be able to operate at ambient pressure, a pre-stage of differential pumping is necessary between the analyzer and the sample environment. Towards the differential pumping system a small aperture limits the gas flow and different aperture sizes can be used to reach different maximum pressures. Presently, the smallest aperture applied here has a diameter of 300 µm and allows pressures up to roughly 5–10 mbar without compromising the UHV at the electron detector, but in the future it can be replaced by even smaller apertures further increasing the high-pressure limit.

This small aperture, however, limits the maximum geometric acceptance of the spectrometer for the incoming electrons. To take full advantage of the incoming photons, the spot size in the sample plane must match this geometric acceptance. In the case of the 300 µm aperture, an optimum detection efficiency is achieved with a photon spot size of about 100 µm × 100 µm (FWHM) at the sample.

The light is let into the AP cell through a thin Si_3_N_4_(200 nm)/Al(100 nm) membrane that provides pressure isolation. However, the transmission of this type of window is reasonable only above approximately 250 eV photon energy, and, in order to facilitate the use of the low energies achievable by the SPECIES beamline, pure Al (thickness 200 nm) windows purchased from Luxel are also available. These give reasonable transmission of approximately 60% from 30 to 70 eV, allowing the studies of the valence bands under ambient-pressure conditions in addition to the core-level spectroscopies. Upstream of the endstation a so-called beam stopper ion pump (manufactured by XIA Inc.) is used, allowing five orders of magnitude higher pressure at the inlet side (experiment) compared with the outlet side. The beam stopper pump together with a turbomolecular pump after the endstation allow experiments with low 10^−4^ mbar pressure in the main chamber of the spectrometer without compromising UHV conditions of the beamline. This concept has been used previously on several beamlines at the MAX IV Laboratory (and MAX-lab) with good results (Aksela *et al.*, 1994 ▸; Bässler *et al.*, 2001 ▸; Urpelainen *et al.*, 2010 ▸; Schnadt *et al.*, 2012 ▸).

The reaction cell gas composition is controlled through a gas manifold system equipped with mass flow controllers, which allow fast switching of gases. The inlet and outlet lines of the reaction cell are connected to a quadrupole mass spectrometer (QMS), which allows monitoring the reactants and the reaction products *in situ* as a function of time simultaneously with the XPS measurements. By connecting the time stamps of the mass spectra and the X-ray photoelectron spectra, the gas composition measured with the QMS can be correlated to changes on the surface chemistry observed using APXPS.

Another new development at the endstation is a centralized backing pump setup. The SPECIES APXPS setup operates 12 permanent turbomolecular pumps. The system is divided into two groups of 5 and 7 turbomolecular pumps. The group of 5 pumps experiences a heavy gas load, while the other group has to cope with a low, UHV-typical, load of gas only. Both groups of pumps are backed by two centralized backing pump systems, each pumped by an ACP 40 multi-stage roots pumps. When UHV conditions are reached, the backing pressure requirements are not extremely stringent and a large backing volume can be used instead of continuous pumping in order to maintain the UHV pressures. This allows the operation of the whole setup with only two backing pumps which can be disconnected for maintenance or replacement, while the turbomolecular pumps on the system are still running. This makes the maintenance of the system cost efficient and reduces the amount of down time significantly. The backing system is controlled by the standard PLC system of MAX IV Laboratory.

The setup includes standard sample preparation and characterization tools such as an electron beam heater for annealing, an ion gun for Ar^+^ sputtering and a low-energy electron diffraction (LEED) setup for surface structure characterization. The system contains a fast-access load lock, a sample preparation chamber and an analysis chamber, which have independent vacuum systems separated by manual gate valves. This allows for sample preparation at high temperatures and varying the gas atmosphere without compromising the UHV conditions of the analysis chamber. Furthermore, the analyzer and reaction cell vacuums can be isolated from the analysis chamber. The load lock can be vented, loaded and evacuated within approximately half an hour, which facilitates fast sample transfers. In addition, the analysis chamber hosts a sample ‘garage’, which can hold up to three samples simultaneously for fast switching between samples during measurements both on the UHV manipulator and in the reaction cell.

Other equipment available at the endstation includes equipment (electrospray setups, evaporators *etc*.) for depositing atoms, molecules and clusters on the surfaces, and equipment (light source, optical fiber *etc*.) for photocatalysis experiments. In addition to the synchrotron, an X-ray source with Al and Mg *K*_α_ anodes is mounted on the system for off-line studies of samples when the RIXS branch is operating and during ring shutdown periods.

Before being installed at MAX IV Laboratory the endstation will be upgraded. The planned upgrades include the construction of a new improved flow cell with a gas flow that is focused onto the target position, a cell and a gas manifold for sulfur-containing and corrosive gases, an electrochemical cell, and the upgrade of the pre-lens of the electron energy analyzer allowing easier and more reliable docking of the reaction cell as well as improved transmission and resolution of the electron analyzer. As a future alternative, the analyzer could be further upgraded to allow imaging measurements.

### 5.4. Atomic layer deposition of SiO_2_ on TiO_2_(110)   

As one example of research carried out at the APXPS endstation of the SPECIES beamline we briefly present some results obtained on the atomic layer deposition (ALD) of SiO_2_ on TiO_2_(110). ALD is a major technique for the controlled deposition of thin films (Miikkulainen *et al.*, 2013 ▸), which builds on the alternating exposure of a substrate to two different precursors. For instance, for the growth of ultrathin oxide layers, metal precursor and oxygen sources are used. In the ideal ALD case, a step-by-step growth is achieved since the adsorption of the precursors on the substrate is self-limited to a single adsorbate layer. This layer can then react with the other precursor.

Although many different ALD processes have been invented, surprisingly little beyond idealized schemes is known about the surface chemistry which takes place during growth and which is decisive for the final film quality. *In situ* techniques applied during growth, *e.g.* APXPS, have the potential to deliver valuable information on this point and thus to help to improve ALD precursors and processes.

**Figure 7:**
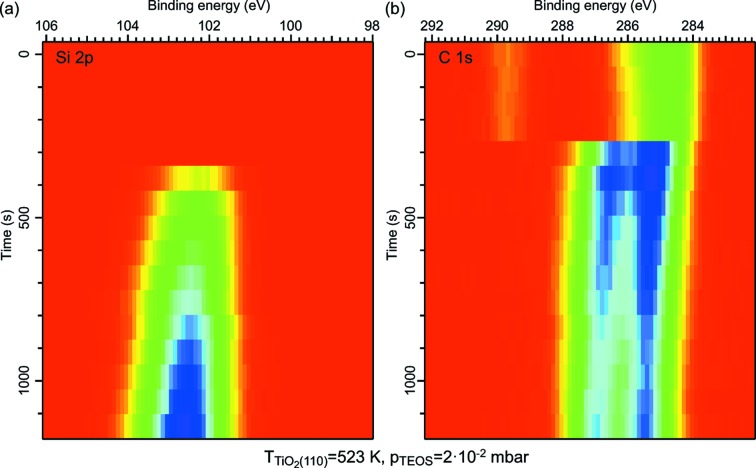
(*a*) Si 2*p* and (*b*) C 1*s* XP spectra series recorded during the exposure of a rutile TiO_2_(110) single-crystal held at 523 K to 2 × 10^−2^ mbar of TEOS. The spectra were measured alternately, and the time stamp is that of the start of the Si 2*p* measurement followed by that of the C 1*s* region. The intensity scale is: blue, high; red, low. The TEOS valve was opened during the initial scans, and the pressure reached the final pressure of 2 × 10^−2^ mbar after around 300 to 400 s, *i.e.* at the same time as the TEOS Si 2*p* and C 1*s* signals appear. The initial C 1*s* intensity is due to residual gas adsorption. The overall decreasing intensity in the C 1*s* spectra is due to the movement of the sample to avoid beam damage. The Si 2*p* spectra were corrected for this intensity loss, but not the C 1*s* spectra due to problems in the data analysis.

For the very first ALD half-cycle of SiO_2_ growth on TiO_2_(110) from tetraethyl orthosilicate [TEOS, Si(OC_2_H_5_O)_4_] and water, *i.e.* for the first exposure of the TiO_2_ surface to TEOS at a pressure of 2 × 10^−2^ mbar, we show the evolution of the Si 2*p* and C 1*s* core levels in Fig. 7 ▸ (for details, see figure caption). Clearly visible is the initial appearance of Si 2*p* (102.29 eV), methyl C 1*s* (285.20 eV) and oxygen-bonded carbon C 1*s* (286.37 eV) features due to the adsorption of TEOS on the surface. During TEOS exposure all core levels shift to higher energy: the Si 2*p* and methyl C 1*s* peaks move to 0.29 eV higher binding energy, and the oxygen-bonded carbon C 1*s* peak shifts by +0.57 eV. In addition, the intensity ratio of the carbon peaks changes slightly. While the overall shift might be due to general band bending, the observation of a deviating shift of the oxygen-bonded carbon peak together with a change of the C 1*s* peak intensity ratio points to the observation of a chemical reaction on the surface. Most likely, ethoxy ligands are split off and bind directly to the surface in a reaction which is unforeseen in the idealized scheme (*cf*. Chaudhary *et al.*, 2015 ▸).

In this example each spectrum, taken in swept mode, required 38 s to be completed. Much improved time resolution even down to the millisecond timescale is possible when the electron energy analyzer is used in snapshot rather than in swept mode. For many surface reactions the attainable time resolution is sufficient to allow following of reaction kinetics and measurement of reaction constants, and APXPS therefore renders possible a field of activity which can deliver information on the evolution of surface (and, actually, gas phase) species in direct combination with kinetic data.

### 5.5. APXPS study of CO oxidation on Ag(100)-supported CO oxide films   

Cobalt oxide nanomaterials have attracted attention because of their application potential in the fields of heterogeneous catalysis (Chen *et al.*, 2015 ▸; Xie *et al.*, 2009 ▸; Fei *et al.*, 2012 ▸). In this example we have studied the CO oxidation reaction on Ag(100)-supported Co oxide films using the APXPS setup at the SPECIES beamline.

**Figure 8:**
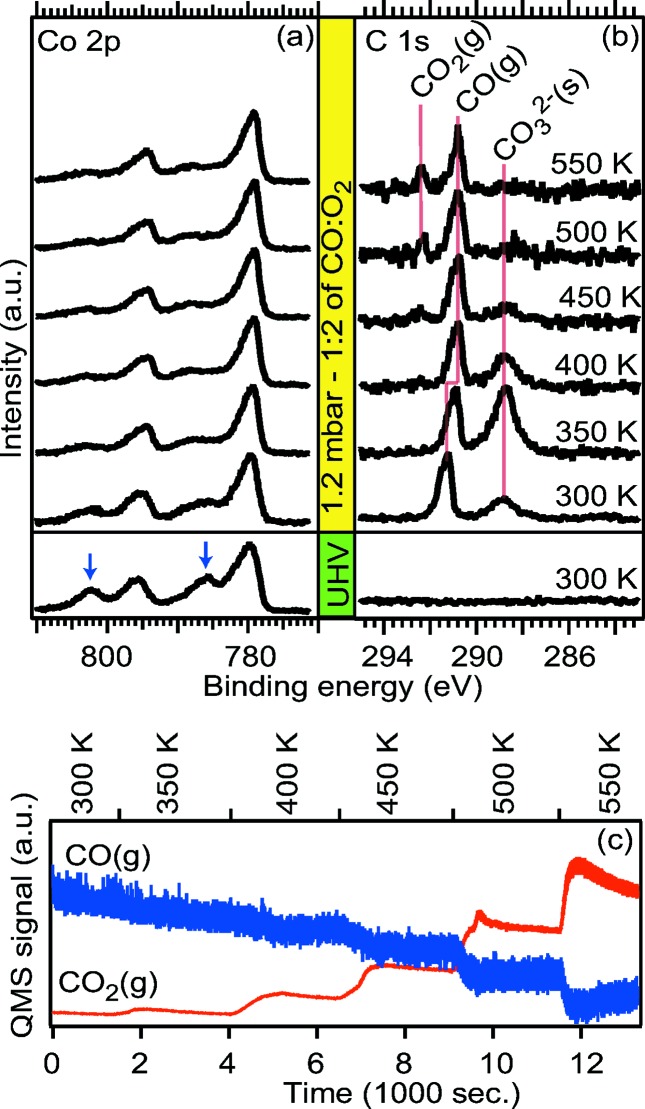
(*a*) Co 2*p* spectra of Ag(100)-supported CoO(100) acquired in UHV at room temperature (bottom) and in 1.2 mbar of a 1:2 CO:O_2_ mixture at stepwise increasing temperature. (*b*) C 1*s* spectra corresponding to the Co 2*p* spectra shown in panel (*a*). (*c*) CO and CO_2_ signals in the exhaust gas from the cell recorded with quadrupole mass spectrometry.

The starting point in the present example is a CoO(100) film grown on Ag(100). The Co 2*p* spectrum shown at the bottom of Fig. 8(*a*) ▸ was recorded under UHV conditions and the shake-up peaks (highlighted with blue arrows) seen at the high binding energy side of the Co 2*p*_3/2_ and Co 2*p*_1/2_ peaks are the fingerprint of the CoO rocksalt phase. In the corresponding C 1*s* spectrum [Fig. 8(*b*) ▸] no carbon-containing species are observed.

Exposing the CoO(100) film to a reaction mixture of a 1:2 mixture of CO:O_2_ at a total pressure of 1.2 mbar while heating we observe that the Co 2*p* shake-up peaks signaling the CoO rocksalt structure gradually disappears upon heating to 400 K [see Fig. 8(*a*) ▸], due to conversion to a spinel Co_3_O_4_(100) film. In the corresponding C 1*s* spectrum recorded at 300 K we observe two peaks located at 288.7 eV and 291.2 eV assigned to carbonates (Ferstl *et al.*, 2015 ▸) and gas-phase CO molecules, respectively. The intensity of the carbonate component first increases upon heating and reaches a maximum at a temperature of 350 K. At higher temperature the intensity of the carbonate component decreases and at 500 K no carbonates are left on the surface. Upon the film conversion from CoO to Co_3_O_4_, which takes place at a temperature between 300 K and 400 K, the CO(g) component shifts to 290.8 eV, which indicates a work function shift between the CoO(100) and Co_3_O_4_(100) surfaces of 0.5 eV, as the binding energies of gas-phase molecules are pinned to the vacuum level which is determined by the surface work function.

Finally, in the C 1*s* spectrum recorded at temperatures of 500 and 550 K we observe a CO_2_ gas-phase component at a position of 292.4 eV. Production of CO_2_ in the gas phase is also visible in the QMS signal recorded simultaneously both at this temperature and lower temperature, which suggests that the Co_3_O_4_(100) phase is catalytically active. Unfortunately, we also observed CO_2_ production in a reference experiment in which a clean Ag(100) crystal was exposed to an identical gas mixture and heating rate and we are, therefore, unable to correlate the observed CO_2_ production to the appearance of the Co_3_O_4_(100) phase. One tentative explanation for the observed CO_2_ production on the clean Ag(100) crystal could be remaining Co oxide on the sample plate left after sputtering.

To conclude, we have shown that Ag(100)-supported CoO(100) thin film is easily converted to Co_3_O_4_(100) in a 1:2 mixture of CO:O_2_ at a total pressure of 1.2 mbar. We observed CO_2_ production both with APXPS directly above the surface and in the QMS attached to the exhaust gas from the cell. It remains, however, unclear whether the observed CO_2_ formation is formed on the Co_3_O_4_(100) surface or at other hot parts inside the AP cell (Nguyen & Tao, 2016 ▸). A final but very important take-home message from our study is, therefore, that reference experiments are essential for correlating observed reactivity to changes on the sample surface. Furthermore, there is a clear need for improved reactivity cells with more localized heating, so that other reactive surfaces near the single-crystal remain cold when the single-crystal is heated. The MAX IV Laboratory is currently developing such cells for their future APXPS endstations.

## 6. Summary   

The SPECIES beamline offers a platform for electron spectroscopy experiments in UHV and ambient-pressure conditions. The second branch is dedicated to resonant inelastic scattering experiments. This unique combination of these two complementary techniques allows the electronic structure of matter to be studied, both at surfaces and in bulk, and both in UHV and at elevated pressures. Furthermore, the beamline reaches low photon energies suitable for valence band studies, not available at other existing APXPS beamlines at other synchrotron radiation facilities. We have shown that the beamline meets the design parameters very well and performs as expected. The beamline is currently being built up at the 1.5 GeV ring at the MAX IV Laboratory, where it is expected to be operational and opened for regular users in early 2018.

## Figures and Tables

**Table 1:** Beamline details The beam size is given as a full with at half-maximum value.

Beamline name	SPECIES
Source type	EPU61
Mirrors	Au-coated
Monochromator	cPGM
Energy range (keV)	0.03–1.5
Wavelength range (Å)	470–8.3
Beam size (µm)	5 × 25 (RIXS) and 100 × 100 (HP-XPS)
Flux (photons s^−1^)	1 × 10^13^–1 × 10^11^, *R* = 10000
